# Prevalence, awareness, attitudes, practices, and associated factors of obesity among adults in Makkah, Saudi Arabia

**DOI:** 10.1186/s42506-025-00189-9

**Published:** 2025-05-01

**Authors:** Enas Alfalogy, Nahla H. Hariri

**Affiliations:** 1 Family Medicine Department, Faculty of Medicine, Suez Canal University, Ismalia, 41511 Egypt; 2 Community Medicine and Pilgrims Healthcare Department, College of Medicine, Umm Al-Qura University, Makkah, 21955 Saudi Arabia

**Keywords:** Obesity, Prevalence, Awareness, Attitude, Practices, KSA

## Abstract

**Background:**

Obesity is becoming increasingly prevalent throughout the world, impairing both life expectancy and quality of life. Despite existing knowledge and awareness about obesity, significant gaps remain in understanding its associated factors and the effectiveness of interventions. This study estimates the prevalence of obesity, identifies its associated factors, and assesses participants’ awareness, attitudes, and practices concerning obesity.

**Methods:**

An analytical cross-sectional study was conducted among 368 adults visiting primary care facilities in Makkah, Saudi Arabia. A structured, pre-validated questionnaire adapted from previous research was used to collect demographic information and assess respondents' awareness, attitude, and practices regarding obesity.

**Results:**

The mean age of the participants was 41.8 ± 2.2 years, and 57.6% were females. Most respondents (97.8%) did not smoke, 32.1% had a family history of obesity, 86.1% consumed an unhealthy diet, and 76.6% did not engage in physical exercise. Approximately 37% of participants were overweight or obese. The study found that 85.9% of participants had a good awareness of obesity, 51.4% demonstrated a favorable attitude, and 33.4% exhibited adequate practices regarding obesity. A considerable proportion of participants (56.3%) recognized obesity based on self-perception. Approximately 38% of participants consumed high-calorie meals when stressed, 24.7% did not get sufficient sleep, and 64.4% did not drink enough water. Most of the participants (91.6%) perceived obesity as a disease, and 60.3% were satisfied with their body shapes. The logistic regression analysis demonstrated that the strongest predictors of obesity were poor awareness of obesity (OR = 10.6, *p* < 0.001), followed by irregular exercise (OR = 6.3, *p* < 0.05), and being female (OR = 4.8, *p* < 0.001). Adequate water intake was found to decrease the likelihood of obesity (OR = 0.1, *p* < 0.001).

**Conclusion:**

Obesity is prevalent among adults in Makkah. Despite a favorable attitude and a good awareness of most aspects relating to obesity, inappropriate practices are common. Additional action and decisions are required to put awareness and attitude into practice.

## Introduction

Increased body fat is the hallmark of obesity, a complex and preventable health issue that has significant consequences on various aspects of health. Ultimately, it leads to a reduced life expectancy and a decreased quality of life. Obesity can develop when calorie consumption exceeds physical activity and is influenced by a combination of environmental, social, lifestyle, and genetic factors. Screening for obesity can be done using various scientific calculations such as body mass index (BMI), waist circumference, and waist-to-hip ratio, with BMI being the most commonly used method [1]. 

Globally, obesity (defined as a BMI of 30 kg/m^2^ or more) is increasingly prevalent across all age groups, especially among young people [2]. According to the World Health Organization (WHO), the global prevalence of obesity tripled between 1975 and 2016. At present, more than 1.9 billion individuals worldwide are overweight, with over 650 million classified as obese. In addition, 213 million children and teenagers fall into the overweight category [3]. A newly released WHO report on the status of obesity in the European region indicates that overweight and obesity rates have reached epidemic levels: 59% of adults and nearly one third of children are affected by overweight or obesity across the region [4]. The prevalence of overweight ranged from 25 to 50% across different countries, including Saudi Arabia, Kuwait, Iran, Lebanon, Qatar, and Jordan. Obesity prevalence varied between 10 and 45%, with higher rates in Gulf Cooperation Council (GCC) countries [5]. The highest estimate for the prevalence rate of obesity in Saudi Arabia is 35.6% and is significantly affected by various social, regional, environmental, dietary, and lifestyle factors [6].

Obesity places a significant burden on both the health and economy of Saudi Arabia. It is a leading risk factor for a range of chronic diseases, including type 2 diabetes, cardiovascular diseases, and certain cancers and its rising prevalence has led to increased mortality and morbidity rates. Studies reveal that nearly 70% of the adult population in Saudi Arabia is either overweight or obese, with obesity being a major driver of preventable deaths due to complications such as heart disease and diabetes [7, 8]. This also places a heavy strain on the healthcare system, as the management of obesity-related conditions requires long-term medical care, including hospital admissions, medications, and surgeries such as bariatric procedures [9]. Economically, the impact of obesity in Saudi Arabia is substantial. The direct healthcare costs associated with treating obesity-related diseases, such as diabetes management and cardiovascular interventions, are significant. Moreover, the indirect costs related to lost productivity, absenteeism, and premature mortality further exacerbate the economic burden.

The worldwide burden of obesity is alarming, as it is responsible for nearly 4.7 million premature deaths per year. It accounted for 8.4% of all global fatalities in 2017 and was the fifth leading preventable cause of death. Obesity also has a high economic burden, as the direct cost of obesity is $3.8 billion, in addition to its impact in other areas. The growing healthcare expenditure in Saudi Arabia, driven by the rising prevalence of obesity and its related diseases, has prompted the government to prioritize public health interventions aimed at curbing obesity rates [10].

In Saudi Arabia, the risk factors for obesity are driven by a combination of dietary changes, physical inactivity, and cultural influences. The shift towards Westernized eating habits, characterized by a high consumption of fast food, sugary beverages, and processed snacks, has contributed significantly to rising obesity rates. Physical inactivity is prevalent, partly due to the hot climate, which discourages outdoor activities, and increased reliance on cars and technology, leading to more sedentary lifestyles [11, 12]. Socioeconomic factors, such as higher income levels and urbanization, have also played a role, as they often correlate with wider access to unhealthy foods and less emphasis on physical activity [13–15]. In addition, cultural norms that associate larger body sizes with wealth and health contribute to overeating, while a lack of comprehensive public health initiatives aimed at promoting exercise and balanced diets further exacerbates the issue [13, 16]. These factors have combined to make obesity a significant public health challenge in the country.

Misperceptions regarding obesity, its prevention, and available management strategies have been observed not only in patients but also among healthcare providers. These misunderstandings can hinder effective counseling and prevent healthcare professionals from adequately guiding patients in managing their obesity and safeguarding their health. This underscores the need for regular surveillance of obesity and other non-communicable diseases, as they further impact key health determinants such as socioeconomic status and physical activity levels [17]. Routine monitoring is essential to address these issues and promote improved health outcomes.

Despite its growing prevalence, there remains a significant gap in understanding the specific factors contributing to obesity in the local context. This study addresses this gap by estimating the prevalence of obesity, identifying its associated risk factors, and assessing the awareness, attitudes, and practices of individuals attending primary healthcare centers (PHCs) in Makkah, Saudi Arabia.

## Methods

### Study design

An analytical cross-sectional study was conducted among 368 adults at PHCs in Makkah, Saudi Arabia who agreed to sign a written informed consent. The study took place between March and June 2023.

### Sample size

The sample size was calculated using a standard cross-sectional calculation [18]: *n* = [*Z*_*α*/2_^2^*P* (1-*P*)]/*d*^2^, where the Z value corresponds to the anticipated level of confidence (*Z* = 1.96 for a 95% confidence interval (CI)), *P* is the probable prevalence (*P* = 0.4 [19]), and d is the precision level (*d* = 0.05). A total of 368 respondents were required to gain a 95% confidence interval.

### Sampling technique

Makkah city is divided into four districts: north, east, west, and south. A cluster random sampling technique was applied, with two PHCs randomly selected from each district, resulting in a total of eight PHCs. A systematic random sample was taken from these centers by selecting every fifth patient from the registration list to ensure a representative sample. The interval was determined based on the total number of patients and the required sample size. Patients were enrolled equally across all centers until the target sample size was achieved. To secure approval for the research, the researcher engaged with PHCs management, providing a comprehensive explanation of the study’s objectives and methodology.

### Study tools

#### Questionnaire

Data were collected using a structured and pre-validated questionnaire to assess factors associated with obesity and the participants'awareness, attitude, and practice related to it. The questionnaire was adapted from previous studies [20, 21] The questionnaire consisted in total of four sections. Section 1 inquired about sociodemographic data and variables linked to obesity, including age, gender, marital status, family history of obesity, chronic illnesses, history of hormone disruptions, smoking, current behaviors, and dietary habits. Section 2 included questions that assessed participants’ awareness of obesity, such as the definition of the normal BMI of an adult, its risk factors, and the burden of obesity on health. Section 3 addressed the participants’ attitudes toward obesity, including perceptions of obesity as a disease and attitudes toward body weight and shape. Section 4 included questions on the participants’ dietary modifications, patterns of water intake, sleep habits, and exercise routines. The awareness, attitude, and practices part of the questionnaire included eighteen items across three categories. The awareness section included seven items, focusing primarily on participants’ awareness of the risk factors and complications related to obesity. The attitude section consisted of four items aimed at evaluating perceptions of obesity and motivation for weight loss. The body weight-related practice component of the tool comprised seven questions about eating habits and physical activity.

The validity of the questionnaire was assessed using content and face validity. Content validity addresses all significant parts of the questionnaire based on previous studies, while face validity is determined by expert judgment. In addition, we conducted a pilot study with a small sample of the target population to ensure its suitability in the current situation, and minor changes were made based on feedback from the pilot study.

The reliability of the questionnaire was analyzed using Cronbach’s *α* analysis, which yielded a value of 0.79 for the questionnaire as a whole. The independent Cronbach’s *α* values for the awareness, attitude, and practices domains were 0.78, 0.77, and 0.70, respectively. The analysis confirmed acceptable internal consistency and reliability.

##### Scoring system for awareness, attitude, and practices towards obesity

The scoring system for evaluating awareness, attitude, and practices related to obesity was structured as follows. Each section’s overall score was calculated by summing the correct responses. Awareness scores were categorized as “good” or “adequate” if they reached or exceeded 75% of the total possible score, whereas scores below this threshold were classified as “poor.” In parallel, an attitude was deemed “favorable” if the score was 75% or higher of the total attitude score. Likewise, practice was considered “adequate” if the score was 75% or higher of the total practice score [22, 23].

#### Anthropometric measurements

Weight and height were used to calculate BMI.

##### Weight and height measurements

The participants’ weights were determined using a properly calibrated digital scale. They were instructed to wear light clothing and stand barefoot and upright on the scale. Height was measured using a calibrated stadiometer, which provides a precise reading. Participants were asked to remove their shoes and stand upright with their heels together, back straight, and head in a neutral position. The measurement was taken to the nearest centimeter, ensuring that the participant was looking straight ahead and not slouching. This standardized approach helped to minimize variability and provided consistent data for analysis. The weight and height were rounded to the closest 0.1 kg (kg) and 0.5 cm (cm), respectively [24].

##### Estimation and classification of body mass index

The BMI was calculated by dividing weight in kg by height in m^2^. The WHO’s classification method was used to categorize BMI and obesity [25]. According to this classification, individuals with a BMI of less than 18.5 kg/m^2^ are considered underweight. Those with a BMI between 18.5 and < 25 kg/m^2^ are classified as having a normal weight. Individuals with a BMI between 25 and < 30 kg/m^2^ are considered overweight. Those with a BMI of ≥ 30 kg/m^2^ are considered obese. Class I obesity includes individuals with a BMI between 30 and < 35 kg/m^2^. Class II obesity includes those with a BMI between 35 and < 40 kg/m^2^. Class III obesity refers to individuals with a BMI ≥ 40 kg/m^2^ [26].

### Statistical analysis

The data were analyzed using the SPSS package, version 25. For presenting sociodemographic characteristics, factors associated with obesity, awareness, attitude, and practices regarding obesity, frequency and percentage were used for categorical variables, and the mean, and standard deviation (SD) were utilized to present quantitative variables. To determine the relationship between sociodemographic and other associated factors and obesity, the chi-square test was used for qualitative variables, and the Student’s *t*-test was used for quantitative variables. In addition, logistic regression was conducted for multivariate analysis to estimate odds ratios (OR), while 95% confidence intervals (CI) were utilized to identify predictors of obesity. Statistical significance was defined as a *p-*value less than 0.05.

## Results

The mean age of the participants was 41.8 years (SD = 2.2). More than half (57.6%) were women. Most respondents (97.8%) did not smoke, 32.1% had a family history of obesity, 10.6% had chronic diseases, and only 1.1% had hormonal problems. Overall, 86.1% consumed an unhealthy diet, 76.6% did not engage in physical exercise, and 60.3% were satisfied with their body shape (Table 1).

**Table 1:** General and lifestyle characteristics of the study sample of adults attending PHCs in Makkah, KSA, 2023 (*n* = 368)

Variable	Frequency (*n*)	Percentage (%)
Age (years)	41.8 ± 2.2	
Gender		
Male	156	42.4
Female	212	57.6
Family history of obesity		
Positive	118	32.1
Negative	250	67.9
Chronic diseases		
Yes	39	10.6
No	329	89.4
Hormonal disturbances		
Yes	4	1.1
No	216	58.7
I don’t know	148	40.2
Smoking		
Yes	8	2.2
No	360	97.8
Doing exercise		
Yes	86	23.4
No	282	76.6
Eating healthy diet		
Yes	51	13.9
No	317	86.1
Perception of body shape		
Satisfied	222	60.3
Dissatisfied	146	39.7

**Fig. 1:**
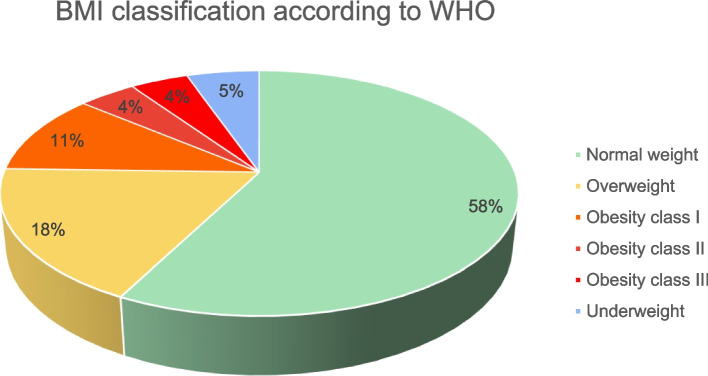
Prevalence of obesity and various BMI classes according to the WHO classification

Figure 1 illustrates the prevalence of obesity and various BMI classes according to the WHO classification. It indicates that 37% of the patients were either overweight or obese (18% were overweight and 19% were obese).

The association between various factors and the prevalence of obesity is presented in Table 2. We found significant relationships between the prevalence of obesity and a positive family history of obesity, having a chronic illness, living a sedentary lifestyle with irregular exercise, smoking, and having inadequate or poor awareness of obesity (i.e., an awareness score of less than 75%). However, having a favorable attitude and being satisfied with one’s body shape were significant in non-obese individuals.

**Table 2:** Factors associated with obesity among the studied adults attending the PHCs in Makkah, KSA, 2023

**Factors associated with obesity**	**Obesity**		**Total** ***n* (%)**	***P* value**
	Yes *n* (%)	No *n* (%)		
Gender				0.000
Male	28 (17.9)	128 (82.1)	156 (42.4)	
Female	43 (20.3)	169 (79.7)	212 (57.6)	
Family history of obesity	59 (42.4)	59 (25.8)	118 (32.1)	0.001
Chronic diseases	28 (20.1)	11 (4.8)	39 (10.6)	0.000
Insufficient exercise	115 (82.7)	167 (72.9)	282 (76.6)	0.020
Inadequate sleep	48 (34.5)	43 (18.8)	91 (24.7)	0.001
Smoking	8 (5.8)	0 (0)	8 (2.2)	0.000
Poor awareness	36 (25.9)	16 (7)	52 (14.1)	0.000
Favorable attitude	32 (23)	157 (68.6)	189 (51.4)	0.000
Adequate practice	95(41.5)	28(20.1)	123(33.4)	0.000
Body shape satisfaction	64 (28.8)	158 (71.2)	222 (60.3)	0.000

**Fig. 2:**
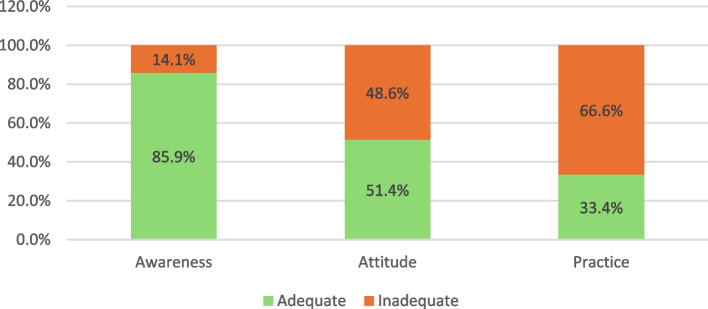
Distribution of frequencies of levels of awareness, attitude, and practices of obesity among participants at PHCsin Makkah

Figure 2 illustrates the distribution of awareness, attitude, and practice classifications among participants. It indicates that 85.9% of participants had adequate awareness, 51.4% had favorable attitudes, and only 33.4% had adequate practices.

### Participants’ awareness of obesity

More than half of the participants (56.3%) recognized obesity based on self-perception rather than scientific calculation, while 75.3% recognized the normal BMI as defined by the WHO or other scientific calculations. The awareness scores ranged from a minimum of 0 to a maximum of 7. The mean and SD of the awareness score was 6.6 ± 1.2, with 14.1% having poor awareness, scoring less than 75% of the total awareness score. Almost all participants (96.7%) were aware that obesity can lead to cardiac problems. In response to the questions on the causes of obesity, 81.5% mentioned hormone issues, 93.5% recognized that obesity occurs when individuals consume more calories than they burn, and 92.4% believed that psychological issues also play a role in dietary choices (Table 3).

**Table 3:** Awareness of obesity among the studied adults attending the PHCs in Makkah, KSA, 2023

Variable	Frequency (*n*)	Percentage (%)
How do you recognize obesity in individuals?		
Self-perception	207	56.3
Scientific calculations (such as BMI, waist-hip ratio, or waist circumference)	161	43.7
Do you know what an adult’s typical BMI is?		
Yes	277	75.3
No	91	24.7
Do you know that obesity might cause cardiac problems?		
Yes	356	96.7
No	12	3.3
Do you know that hormonal disturbances such as hypothyroidism can result in obesity?		
Yes	300	81.5
No	68	18.5
Do you know that obesity develops when people consume more calories than they burn?		
Yes	344	93.5
No	24	6.5
Do you know that psychological issues also affect dietary choices and obesity?		
Yes	340	92.4
No	28	7.6
Do you know that those who consume junk food are more likely to become obese?		
Yes	360	97.8
No	8	2.2

### Attitude toward obesity among participants at PHCs in Makkah

Approximately 91.6% of participants perceived obesity as a disease and not only a risk factor. Overall, 60.3% were satisfied with their body shape. Participants were asked about their attitude towards obesity, and 51.4% believed that having a normal weight is crucial for optimal health. Moreover, 67.9% stated that maintaining an appropriate weight is always difficult, and 44.8% believed that increased weight is harmful to health. The attitude scores ranged from a minimum of 0 to a maximum of 8 (Table 4).

**Table 4:** Attitudes towards obesity among the studied adults attending the PHCs in Makkah, KSA, 2023

Variable	Frequency (*n*)	Percentage (%)	
Do you think obesity is a disease?			
Definitely	337		91.6
Probably	12		3.2
No	19		5.2
Do you consider increased weight harmful to your health?			
Definitely	165		44.8
Probably	135		36.7
No	68		18.5
Is it crucial to have a normal weight to maintain optimal health?			
Definitely	189		51.4
Probably	88		36.6
No	44		12.0
Do you find it difficult to maintain an appropriate weight?			
Always	250		67.9
Sometimes	68		18.5
Rarely	50		13.6

### Body weight-related practices of participants attending PHCs in Makkah

The body weight-related practices of participants are presented in Table 5. About 39% of respondents rarely check their BMI (less than 2 times per month), less than half (44.6%) regularly modify their diet to maintain health, and 40.2% regularly modify their daily activity to preserve their body shape. Only 6.5% of participants eat junk food daily. Approximately 38% consume high-calorie meals during stressful situations and 24.7% sleep for fewer than 8 h daily. The average daily water intake is 7.4 cups, with an SD of 4 cups, and 64.4% of people do not drink enough water (fewer than 8 cups daily). The practice scores ranged from a minimum of 12 to a maximum of 25. The mean and SD of the practice score is 19.1 ± 2.9, with 66.6% having inadequate practice, scoring less than 75% of the total practice score (Table 5).

**Table 5:** Body weight-related practices of the studied adults attending the PHCs in Makkah, KSA, 2023

Variable	Frequency (*n*)	Percentage (%)
Frequency of measuring body weight		
Every time (2 times or more per week)	20	5.4
Most of the time (weekly)	141	38.3
Sometimes (2 to 3 times per month)	63	17.1
Very rarely (less than 2 times per month)	144	39.2
Frequency of making dietary modifications to maintain health		
Every time	20	5.4
Most of the time	164	44.6
Sometimes	148	40.2
Very rarely	36	9.8
Frequency of modifying daily activity to preserve body shape		
Every time	47	12.8
Most of the time	148	40.2
Sometimes	95	25.8
Very rarely	78	21.2
Frequency of eating junk food		
Daily	24	6.5
Weekly	127	34.5
Monthly	217	59.0
Do you eat large meals^a^ during periods of stress?		
Yes	140	38.0
No	228	62.0
Do you drink adequate amounts of water daily?		
< 8 cups/day	237	64.4
≥ 8 cups/day	131	35.6
Do you get enough sleep daily?		
< 8 h/day	91	24.7
≥ 8 h/day	277	75.3

^a^A large meal is one that is high in calories or rich in fat and carbohydrates

### Predictors of obesity among participants attending PHCs in Makkah

Table 6 presents the findings of the binary logistic regression analysis. Those with an obesity-positive family history were more likely to be obese (OR 3.94, 95% CI: 1.83–8.48), females (OR 4.83, 95% CI: 2.05–11.38), with a sedentary lifestyle (OR 6.30, 95% CI: 2.41–16.50), with inadequate sleep (OR 2.29, 95% CI: 1.05–4.99), consume junk food regularly (OR 3.36, 95% CI: 1.06–10.68), dissatisfied with their body shape (OR 3.78, 95% CI: 2.68–6.84), and with a poor awareness of obesity (OR 10.57, 95% CI: 4.39–25.44). The strongest predictors of obesity were poor awareness of obesity, followed by irregular exercise and being female. An adequate water intake decreases the likelihood of obesity.

**Table 6:** Binary logistic regression analysis for predictors of obesity

Predictors of obesity	Significance	OR	95% CI lower	95% CI upper
Family history of obesity	0.00*	3.940	1.830	8.484
Gender (female)	0.000*	4.836	2.053	11.388
Irregular exercising	0.027*	6.306	2.410	16.503
Inadequate sleep	0.036*	2.296	1.056	4.994
Adequate water intake	0.000*	0.105	0.030	0.369
Regular eating of junk food	0.039*	3.369	1.062	10.684
Body shape dissatisfaction	0.000*	3.785	2,685	6.849
Poor knowledge	0.000*	10.576	4.395	25.449

* *p* < 0.05; *OR* odds ratio, *CI* confidence interval

## Discussion

Obesity is a significant health challenge that affects nearly one-third of the global population, imposing a substantial burden due to its predictable health consequences. In this study, 18% of participants were classified as overweight and 19% as obese—figures that are lower than those reported in a nationwide cross-sectional survey in Saudi Arabia, which indicated an obesity prevalence of 21.7% [27]. Moreover, these results are significantly lower than those of a study conducted in Cairo, Egypt which found that 46% of the participants were either overweight or obese [28]. According to the UAE National Diabetes and Lifestyle Study conducted by Sulaiman et al., the obesity rate in the United Arab Emirates in 2017 stood at 32.3%. The study highlighted the prevalence of overweight and obesity among expatriates, reflecting a significant public health concern in the region [29]. This decline in obesity rates in Saudi Arabia may be attributed to recent policy changes that promote healthier lifestyles. As part of the Vision 2030 initiative, the government has introduced various quality-of-life programs to encourage physical activity and foster a healthier way of living.

The participants in this study demonstrated high levels of awareness and favorable attitudes regarding obesity, yet their practices relating to weight management were found to be inadequate. Understanding individuals’ awareness and attitude toward obesity and weight loss is therefore essential. In this study, 56.3% of participants identified obesity based on self-perception rather than scientific calculations, while 75.3% were aware of the normal BMI, as defined by the WHO. This is in line with the findings of another study conducted in primary care in Medina in which approximately 56% of respondents described obesity as an excessive accumulation of fat in the body – notwithstanding the different methods used for defining obesity – and 60.3% agreed that BMI is a useful measure for determining obesity [30]. Participants in the current research also exhibited a good awareness of the risk factors associated with obesity and its adverse effects. These results are consistent with a study conducted in China, which reported that over 80% of respondents had a good understanding of the common causes and complications of obesity [31]. While this heightened awareness may be attributed to the increased availability of information sources, there is still a need for initiatives that translate awareness and favorable attitudes into effective practices.

As far as attitudes are concerned, 91.6% of respondents recognized obesity as a medical condition. This reflects a significant improvement from a previous study conducted in Saudi Arabia in which 25% of participants did not perceive obesity as a medical illness and believed it to be an inherited condition that could not be managed [32]. In this study, 6.5% of the participants reported eating junk food every day, while 23.4% exercised regularly, which is lower than the findings of a study carried out among respondents from PHCs in Medina. The latter study reported that 87.3% ate fast food and 94% exercised regularly [30]. The low level of physical activity identified by Baig et al. [32] is in line with other studies in Pune and Karnataka in India, which found that 47.7% and 63.5% of the study population, respectively, performed inadequate physical activity, even though exercise significantly contributes to both short-term and long-term weight maintenance [33]. Certain harmful lifestyle choices coexist, interact, and increase the probability of being diagnosed as overweight or obese. Despite having a good awareness of most aspects, as well as a favorable attitude, participants in our study still exhibited unhealthy practices. This discrepancy has also been reported in other studies [34–36]. It would be beneficial to begin educating the younger generation about the harmful effects of obesity and ways to prevent it [37]. This way, they can develop healthy habits and make changes to their lifestyle, eating habits, and sedentary behavior. The multivariate regression analysis results indicated that insufficient awareness of obesity and irregular exercise were the two main predictors of obesity. Factors that increase the likelihood of obesity include being female, leading a sedentary lifestyle, following an unhealthy diet, and expressing dissatisfaction with body image. Similar findings were reported in Malaysia, where individuals who were dissatisfied with their appearance, sedentary, smokers, and at risk of poor dietary habits also exhibited a higher likelihood of obesity [38]. Research has similarly indicated that individuals with a sedentary lifestyle are at greater risk of obesity. In addition, dissatisfaction with body image can lead to eating disorders such as bulimia and binge eating, which may contribute to the development of obesity [39]. Dissatisfaction with body image can also lead to depressive symptoms such as heightened appetite and overeating. Moreover, the easy availability of junk food presents challenges. Therefore, promoting healthy eating has been proposed as an effective strategy to counteract misleading food marketing tactics and encourage consumers to make healthier, less harmful choices.

### Study limitations

Since this research was conducted in Makkah, it is not possible to generalize the results to other locations. Attitude is the only variable reflecting participants’ opinions in the study, unlike awareness and practices, which reveal participants’ information and behavior respectively. Data collection was based on participants’ interview reports, which may subject the data to interviewer and response biases (social desirability). No causal implications could be drawn from the cross-sectional research design, which was limited to examining the correlation between obesity and its associated factors. Sampling from the primary healthcare context may impact the generalizability of the results, as participants'self-reports of awareness, attitude, and practices may lead to biased outcomes.

## Conclusion

The prevalence of overweight and obesity among adults remains a significant concern. Despite a good awareness base and favorable attitude towards obesity, unhealthy practices persist among the participants. Therefore, instead of focusing exclusively on changing public perceptions, efforts should be directed toward improving self-regulation and self-management skills, as well as enhancing self-efficacy. Creating supportive environments for effective weight management is crucial, including providing accessible and affordable facilities for jogging and sports. Moreover, it is recommended that further research be conducted in other regions of Saudi Arabia to gain a better understanding of the national scale of obesity-related issues.

## Data Availability

The datasets used and/or analyzed during the current study are available from the corresponding author upon reasonable request.

## Abbreviations

BMI: Body Mass Index
WHO: World Health Organization
OR: Odds Ratio
CI: Confidence Interval
SD: Standard Deviations
PHCCs: Primary Health Care Centers
